# Biomaterials in Medical Applications

**DOI:** 10.3390/polym15040847

**Published:** 2023-02-08

**Authors:** Hsiuying Wang

**Affiliations:** Institute of Statistics, National Yang Ming Chiao Tung University, Hsinchu 30010, Taiwan; wang@stat.nycu.edu.tw

Natural biomaterials are materials extracted from living organisms or their by-products. These materials have many advantages, such as non-toxicity, biocompatibility, and biodegradability, the use of natural biomaterials has steadily increased due to the high demand for medical applications. These materials, including collagen, chitosan, and alginate, have been successfully used as hydrogels, scaffolds, matrices, and implants in tissue engineering, wound management, drug delivery, and nanotechnology. Due to their biocompatible and biodegradable properties, the use of natural biomaterials may lead to cost savings in healthcare because they can be safely absorbed by the body without additional surgeries. However, biodegradability is also a disadvantage of natural materials because they may not be as durable as traditional synthetic materials and can easily suffer from wear and tear due to intensive interaction with the body. Despite these limitations, the biocompatibility and non-toxicity of natural biomaterials can outweigh their biodegradability disadvantages in many medical applications. Therefore, more studies are needed to investigate the effect of biomaterial treatment and to develop methods to control the degradation rate of these materials.

This Special Issue collects original contributions, both research papers and reviews, showing recent advances in the medication application of biomaterials.

Collagen, commonly found in the extracellular matrix of various tissues, has various medical applications. Igielska-Kalwat et al. [1] developed a hair care product in the form of a gel made from natural collagen obtained from fish waste, and this collagen laminate may eliminate excessive sebum production, restore the appropriate pH value, and reduce skin inflammation. The use of porous sponge-like collagen scaffolds in tissue engineering was examined by Bacakova et al. [2]. The scaffolds were prepared by freeze-drying and then mineralized in a simulated body fluid. The mineralized scaffolds might be more promising for bone tissue engineering than unmineralized scaffolds. Vallecillo et al. [3] investigated the degradation of three collagen matrices (Fibro-Gide, Mucograft, and Mucoderm) over time through different tests. The results showed that Fibro-Gide had the highest resistance to degradation over time in comparison to Mucograft and Mucoderm. A systematic review and meta-analysis were conducted to compare the efficacy of collagen matrices to autogenous connective tissue graft in improving soft tissue dimensions around teeth and implants [4]. The effects of collagen treatment on various diseases and conditions, including skin regeneration, bone defects, sarcopenia, wound healing, dental therapy, gastroesophageal reflux, osteoarthritis, and rheumatoid arthritis, were reviewed by Wang [5]. This review concluded that collagen-based medication might be a good choice for treating multiple conditions, including COVID-19, and preventing complications.

Three-dimensional printing is a rapidly growing field in biomaterials that can be used to create customized scaffolds for tissue engineering, implantable devices, and drug delivery systems. The use of customized biocompatible and bioresorbable polymer-based composite filaments, made by combining polylactic acid (PLA), polycaprolactone (PCL), and hydroxyapatite (HA) through extrusion for 3D printing in the treatment of bone defects was investigated by Akerlund et al. [6]. Hata et al. [7] developed a novel PMMA-based resin for 3D printing by mixing methyl methacrylate (MMA), ethylene glycol dimethacrylate (EGDMA), and PMMA powder in various mixing ratios. The printability and viscosity of the PMMA-based resins were examined to determine their suitability for 3D printing. Zu et al. [8] developed a 3D-printable hydrogel that mimicked the hierarchical structure of the extracellular matrix by embedding nanogels, small particles with versatile chemical and biological properties, into a larger hydrogel called gelatin methacryloyl (GelMA). This new type of hydrogel has the potential to be used in 3D cell cultures and other biomedical applications.

Lee et al. [9] developed a new type of biodegradable vascular scaffold (BVS) made from poly(L-lactic acid) (PLLA) by fabricating PLLA films as the surface of BVS and dividing PLLA films into two coating layers. The modification of PLLA films was expected to have multifunctional abilities to overcome problems of BVS effectively, such as X-ray penetrability, inflammation, thrombosis, and neointimal hyperplasia. The use of lactobionic acid (LBA) mixed with the protein zein and oleic acid as a drug delivery film system was investigated by Coroli et al. [10]. The LBA released from the films was found to have antioxidant properties and inhibit the growth of certain bacteria. The most recent studies in biomaterial dressings and scaffolds suitable for wound management were reviewed by Niculescu and Grumezescu [11]. Recent advances in chemically modified and hybrid carrageenan-based platforms for drug delivery, wound healing, and tissue engineering were reviewed by Mokhtari et al. [12].

The use of pectin, a plant-derived heteropolysaccharide, as a biomaterial for assisting anastomotic healing after intestinal surgery was investigated by Zheng et al. [13]. Pectin showed significantly stronger adhesion to the serosa compared to both nanocellulose fiber films and pressure-sensitive adhesives. The use of polymers as a bone substitute for the treatment of periodontal infrabony defects was reviewed by Onisor et al. [14]. The importance of intraoperative hemostasis in cardiovascular surgery and various hemostatic agents with different properties and mechanisms of action were reviewed by Moldovan et al. [15]. A hydrogel developed by synthesizing Kefiran with methacrylic anhydride for tissue engineering applications was characterized by its physical and chemical properties [16]. Artificial corneas with different shapes (spherical, aspherical, and biconic shapes) were designed, and the artificial corneas could efficiently decrease the imaging blur [17].

Biomaterials development plays a crucial role in advancing medical technology and improving patient outcomes. As the editor of this Special Issue, I am confident that this Special Issue will contribute to research within this field by providing readers with a comprehensive and up-to-date understanding of this topic.

## References

[1] Igielska-Kalwat J., Kilian-Pieta E., Poloczanska-Godek S. (2022). The Use of Natural Collagen Obtained from Fish Waste in Hair Styling and Care. Polymers.

[2] Bacakova L., Novotna K., Hadraba D., Musilkova J., Slepicka P., Beran M. (2022). Influence of Biomimetically Mineralized Collagen Scaffolds on Bone Cell Proliferation and Immune Activation. Polymers.

[3] Vallecillo C., Toledano-Osorio M., Vallecillo-Rivas M., Toledano M., Osorio R. (2021). In Vitro Biodegradation Pattern of Collagen Matrices for Soft Tissue Augmentation. Polymers.

[4] Vallecillo C., Toledano-Osorio M., Vallecillo-Rivas M., Toledano M., Rodriguez-Archilla A., Osorio R. (2021). Collagen Matrix vs. Autogenous Connective Tissue Graft for Soft Tissue Augmentation: A Systematic Review and Meta-Analysis. Polymers.

[5] Wang H. (2021). A Review of the Effects of Collagen Treatment in Clinical Studies. Polymers.

[6] Akerlund E., Diez-Escudero A., Grzeszczak A., Persson C. (2022). The Effect of PCL Addition on 3D-Printable PLA/HA Composite Filaments for the Treatment of Bone Defects. Polymers.

[7] Hata K., Ikeda H., Nagamatsu Y., Masaki C., Hosokawa R., Shimizu H. (2021). Development of Dental Poly(methyl methacrylate)-Based Resin for Stereolithography Additive Manufacturing. Polymers.

[8] Zu G.Y., Meijer M., Mergel O., Zhang H., van Rijn P. (2021). 3D-Printable Hierarchical Nanogel-GelMA Composite Hydrogel System. Polymers.

[9] Lee H.I., Heo Y., Baek S.W., Kim D.S., Song D.H., Han D.K. (2021). Multifunctional Biodegradable Vascular PLLA Scaffold with Improved X-ray Opacity, Anti-Inflammation, and Re-Endothelization. Polymers.

[10] Coroli A., Romano R., Saccani A., Raddadi N., Mele E., Mascia L. (2021). An In-Vitro Evaluation of the Characteristics of Zein-Based Films for the Release of Lactobionic Acid and the Effects of Oleic Acid. Polymers.

[11] Niculescu A.G., Grumezescu A.M. (2022). An Up-to-Date Review of Biomaterials Application in Wound Management. Polymers.

[12] Mokhtari H., Tavakoli S., Safarpour F., Kharaziha M., Bakhsheshi-Rad H.R., Ramakrishna S., Berto F. (2021). Recent Advances in Chemically-Modified and Hybrid Carrageenan-Based Platforms for Drug Delivery, Wound Healing, and Tissue Engineering. Polymers.

[13] Zheng Y.F., Pierce A.F., Wagner W.L., Khalil H.A., Chen Z., Funaya C., Ackermann M., Mentzer S.J. (2021). Biomaterial-Assisted Anastomotic Healing: Serosal Adhesion of Pectin Films. Polymers.

[14] Onisor F., Bran S., Mitre I., Mester A., Voina-Tonea A., Armencea G., Baciut M. (2021). Polymer-Based Bone Substitutes in Periodontal Infrabony Defects: A Systematic Evaluation of Clinical Studies. Polymers.

[15] Moldovan H., Antoniac I., Gheorghita D., Safta M.S., Preda S., Broasca M., Badila E., Fronea O., Scafa-Udriste A., Cacoveanu M. (2022). Biomaterials as Haemostatic Agents in Cardiovascular Surgery: Review of Current Situation and Future Trends. Polymers.

[16] Radhouani H., Correia S., Goncalves C., Reis R.L., Oliveira J.M. (2021). Synthesis and Characterization of Biocompatible Methacrylated Kefiran Hydrogels: Towards Tissue Engineering Applications. Polymers.

[17] Ma Y.C., Hsieh C.T., Lin Y.H., Dai C.A., Li J.H. (2021). Numerical Study of Customized Artificial Cornea Shape by Hydrogel Biomaterials on Imaging and Wavefront Aberration. Polymers.

